# Locking plate fixation versus intramedullary nail fixation for the treatment of multifragmentary proximal humerus fractures (OTA/AO type 11C): a preliminary comparison of clinical efficacy

**DOI:** 10.1186/s12891-023-06567-8

**Published:** 2023-06-05

**Authors:** Minghui Wang, Xiuhui Wang, Pan Cai, Shengyang Guo, Beigang Fu

**Affiliations:** Department of Orthopedics, Shanghai University of Medicine & Health Sciences Affiliated to Zhoupu Hospital, Shanghai, 201318 China

**Keywords:** Clinical efficacy, OTA/AO type 11C1.1 and 11C3.1, Proximal humerus neck fractures, Locking plate, Intramedullary nail

## Abstract

**Background:**

This study aimed to compare the clinical efficacy of locking plate and intramedullary nail fixations in the treatment of patients with OTA/AO type 11C proximal humerus fractures.

**Methods:**

We retrospectively analyzed the data of patients with OTA/AO type 11C1.1 and 11C3.1 proximal humerus fractures who underwent surgery at our institution from June 2012 to June 2017. Perioperative indicators, postoperative morphological parameters of the proximal humerus, and Constant–Murley scores were evaluated and compared.

**Results:**

Sixty-eight patients with OTA/AO type 11C1.1 and 11C3.1 proximal humerus fractures were enrolled in this study. Overall, 35 patients underwent open reduction and plate screw internal fixation, and 33 patients underwent limited open reduction and locking of the proximal humerus with intramedullary nail internal fixation. The total cohort had a mean follow-up duration of 17.8 months. The mean operation time of the locking plate group was significantly longer than that of the intramedullary nail group (*P* < 0.05), while the mean bleeding volume was significantly higher in the locking plate group than that in the intramedullary nail group (*P* < 0.05). The initial neck–shaft angles, final neck–shaft angles, forward flexion ranges, or Constant–Murley scores did not show significant differences between the two groups (*P* > 0.05). Complications, including screw penetrations, acromion impingement syndrome, infection, and aseptic necrosis of the humeral head, occurred in 8 patients (8/35, 22.8%) in the locking plate group and 5 patients in the intramedullary nail group (5/33, 15.1%; including malunion and acromion impingement syndrome), with no significant difference between the groups (*P* > 0.05).

**Conclusions:**

Similar satisfactory functional results can be achieved with locking plates and intramedullary nailing for OTA/AO type 11C1.1 and 11C3.1 proximal humerus fractures, with no significant difference in the number of complications between these two techniques. However, intramedullary nailing has advantages over locking plates for OTA/AO type 11C1.1 and 11C3.1 proximal humerus fractures in terms of operation time and bleeding volume.

## Background

Proximal humerus fractures, particularly anatomical neck fractures in older adults, are prone to failure of internal fixation, commonly resulting in poor prognosis and shoulder function [1, 2]. Patient factors, such as reduced local bone density [3, 4], incomplete medial calcar support [5], and humeral head ischemia [6, 7], may all precipitate these failures, in addition to surgeon-related factors, including inadequate fracture reduction and postoperative displacement [8, 9]. OTA/AO type C proximal humerus fractures remain particularly challenging to treat due to these above-mentioned factors, in addition to difficulties in managing bone voids that remain after fracture reduction [10, 11].

Locking plate fixation remains the gold standard for the treatment of proximal humerus fractures [12, 13]. However, the use of locking plates in the treatment of proximal humerus fractures carries a high risk of complications, such as humeral head varus, screw penetrations, and internal fixation loosening [14, 15]. On the contrary, proximal humerus locking intramedullary nails have become increasingly popular with orthopedic physicians because of their minimally invasive insertion and good stability [16, 17]. Currently, there are no studies comparing the efficacy and safety of locking plate and intramedullary nail fixations in the treatment of OTA/AO type 11C1.1 and 11C3.1 proximal humerus fractures. To address this knowledge gap, we conducted a retrospective analysis comparing the clinical efficacy of locking plate and intramedullary nail fixations for OTA/AO type 11 C proximal humerus anatomical neck fractures.

## Methods

### Patients

We retrospectively analyzed the data collected from patients with proximal humerus fractures who underwent surgical treatment in our hospital from June 2012 to June 2017. The inclusion criteria were as follows: (1) patients older than 18 years of age who consented to undergo surgery, and those able to actively cooperate with functional rehabilitation exercises after surgery; (2) patients with a good general condition who were able to tolerate anesthesia and surgery; and (3) patients treated for OTA/AO type 11C1.1 and 11C3.1 proximal humerus neck fractures. The exclusion criteria were as follows: (1) patients with a preoperative rotator cuff injury with shoulder joint dysfunction, (2) patients with preoperative osteoarthritis of the shoulder joint, and (3) patients with a historical proximal humerus fracture. This study was approved by the Shanghai University of Medicine & Health Sciences Affiliated to Zhoupu Hospital Ethics Committee. All procedures were carried out in accordance with the relevant guidelines and regulations. Written informed consent was obtained from all patients for participation in this study.

A retrospective review of 317 electronic medical records was conducted. Overall, 68 patients were included after applying the inclusion and exclusion criteria, of whom 35 underwent locking plate fixation and 33 underwent intramedullary nail fixation. Patients were divided into two groups according to the type of surgery. Demographic forms, investigating age, sex, and fracture type, were filled out for each patient.

### Surgical procedure

Sixty-eight patients were operated on by two surgeons equally proficient in both methods. The affected limb was treated with cefuroxime half an hour prior to surgery to prevent infection. The choice of locking plates or intramedullary nails depended on the preoperative mechanism of fracture injury and the patient’s bone density. In patients with a varus fracture of the proximal humerus, intramedullary nail fixation was considered first, while locking plate internal fixation was initially considered if the patient had a valgus fracture of the proximal humerus.

**Fig. 1:**
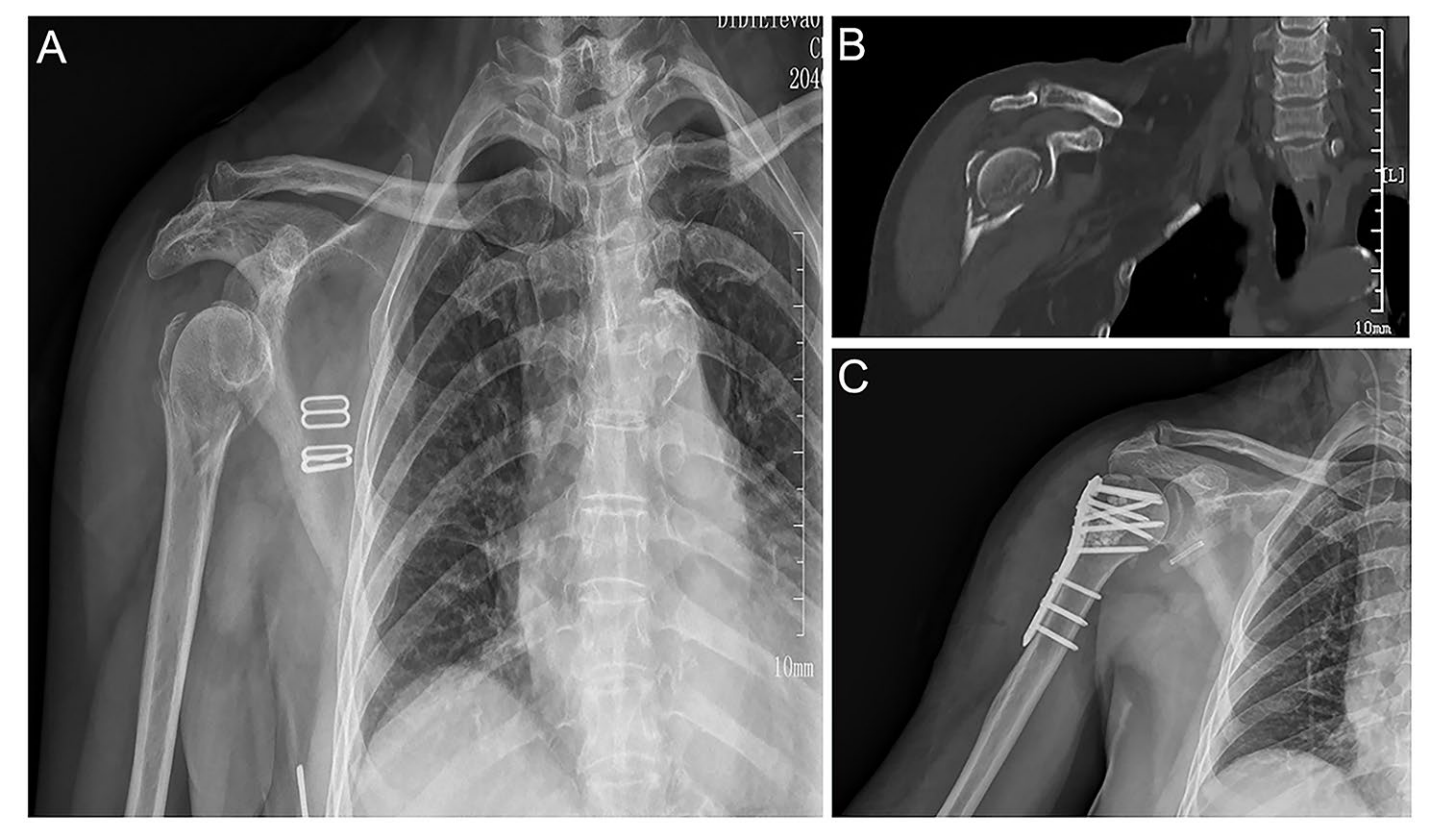
An example case of a right proximal humerus fracture in a 65-year-old woman treated with locking plate internal fixation. **(A)** X-ray images showing the fractures in the anatomical neck of the right humerus and the greater tuberosity, as well as valgus cottage and greater tuberosity displacement; **(B**) CT scan of the fracture and displacement; **(C)** Anterior posterior X-ray image showing good reduction of the anatomical neck and greater tuberosity fracture. CT, computed tomography

**Fig. 2:**
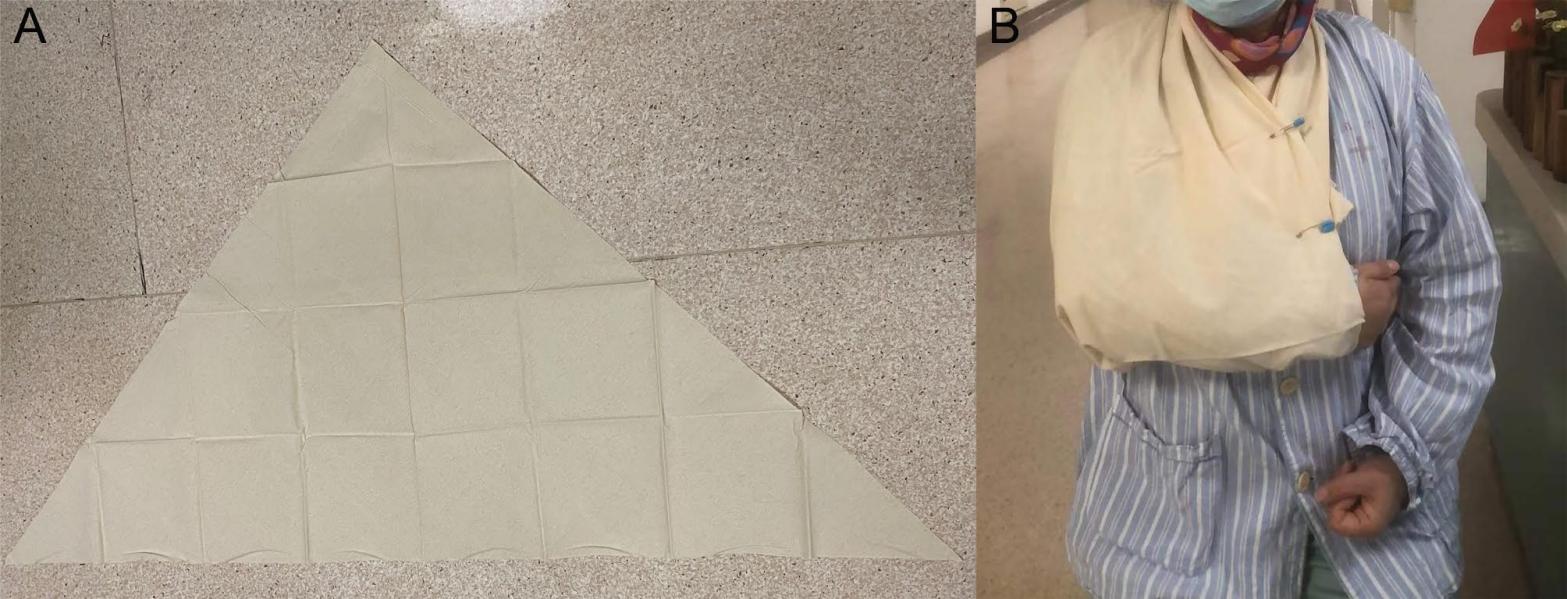
**(A)** A photograph of a triangular towel; **(B)** A picture of a patient hanging with a triangle towel after surgery

For locking plate internal fixation, patients were administered general anesthesia, placed in the supine position, and routinely disinfected. The deltopectoral approach was used in the locking plate group, as previously described [18]. In short, cortical screws were used to secure the plate to the humeral shaft prior to placement of the plate, and proximal humerus locking screw fixation was subsequently completed. C-arm X-ray machine fluoroscopy was further applied to confirm that the fracture reduction and internal fixation were in a good position (Fig. 1). The rotator cuff stitching was then reinforced. The patient was hung with a triangle towel after completion of the surgery (Fig. 2).

**Fig. 3:**
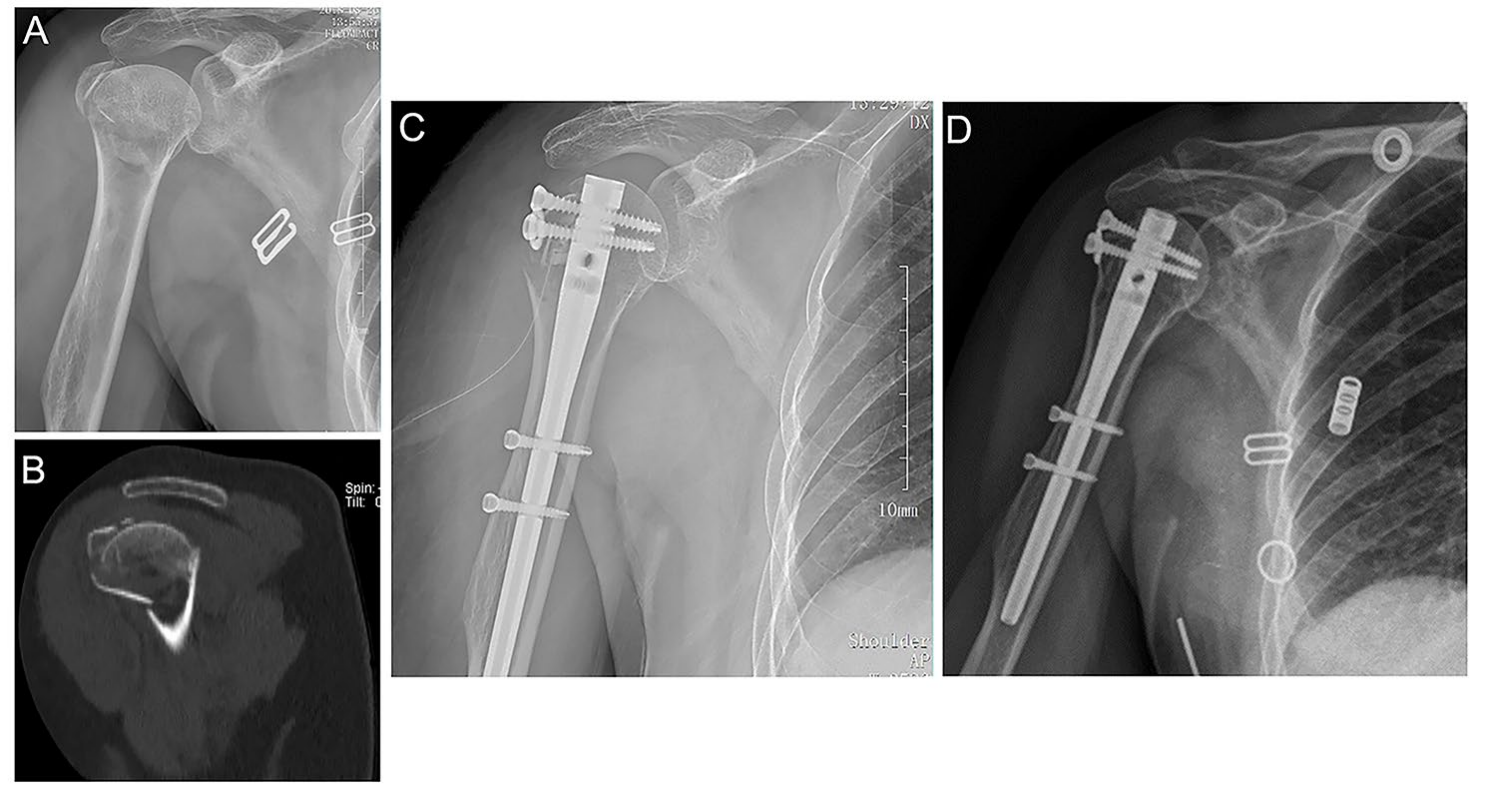
An example case of a right proximal humerus fracture in a 54-year-old woman treated with intramedullary nail fixation. **(A)** X-ray image showing the fractures in the anatomical neck of the right humerus and the greater tuberosity, as well as valgus cottage and greater tuberosity displacement; **(B)** CT scan of the fracture and displacement; **(C)** Postoperative anteroposterior X-ray image showing good reduction of the anatomical neck and greater tuberosity fracture. The proximal and distal interlocking screws satisfactorily positioned; **(D)** Anteroposterior X-ray image of the right humerus anatomical neck and greater tuberosity showed good healing, no loosening of internal fixation, and no displacement of fracture at one year postoperatively. CT, computed tomography

**Fig. 4:**
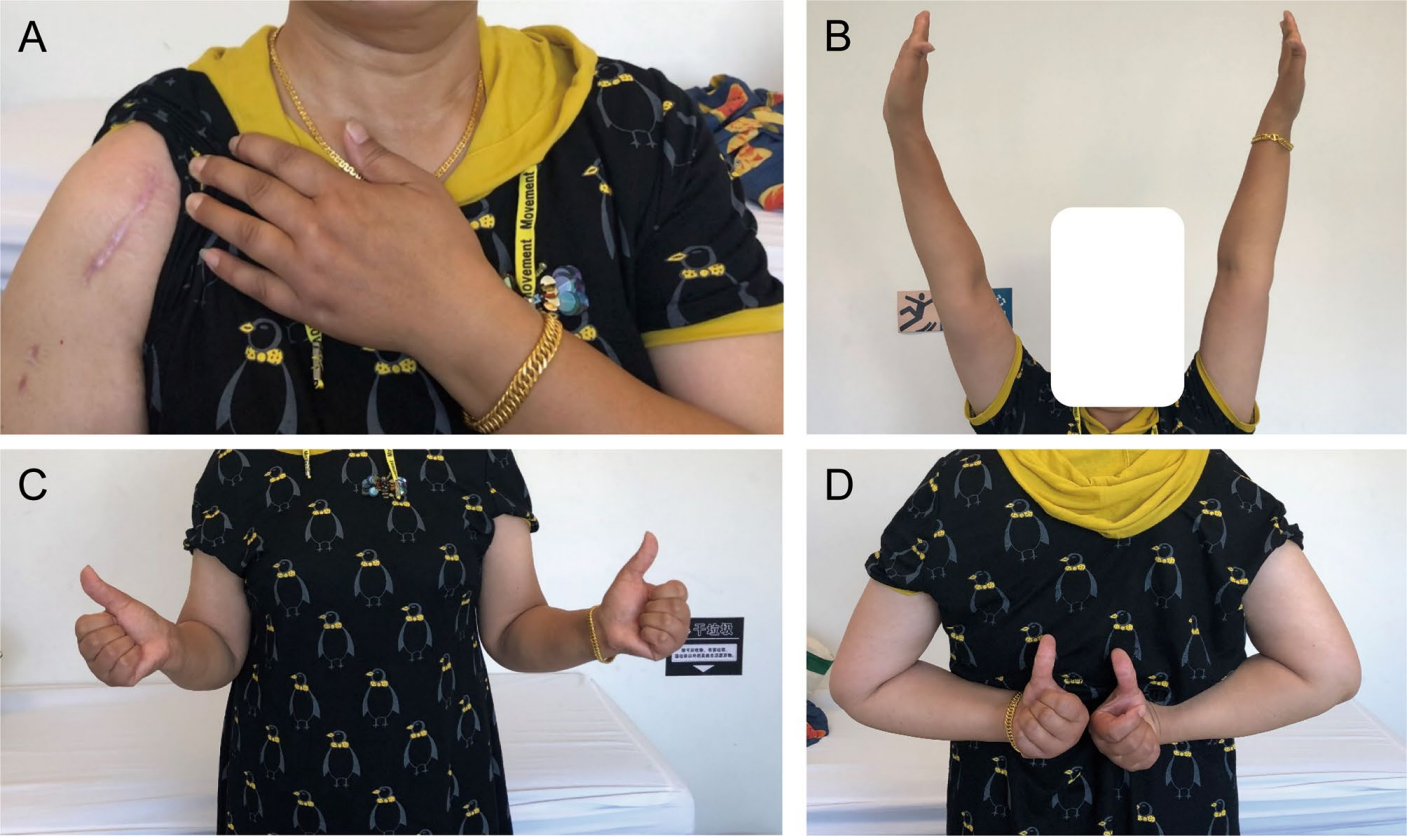
Photographs of the incision at one year postoperatively **(A)**. The function of forward flexion **(B**), external rotation **(C)**, and internal rotation **(D)** recovered well

For intramedullary nail fixation, patients were administered general anesthesia, placed on the beach chair position, and routinely disinfected. A deltoid-splitting approach was used, as previously described [19]. In short, the proximal end of the intramedullary nail was opened, and the TriGen straight nail (Xerox, USA) was inserted after reduction of the fracture. The nail was advanced 1–2 mm into the subchondral bone, the tuberosities were reduced, and screws and sutures were used to strengthen the fixation (Figs. 3 and 4), with proximal fixation performed with 2–4 screws and distal fixation with 1–2 screws. Finally, the wound was washed, sutured layer-by-layer, and the patient was hung with a triangle towel after the operation.

### Postoperative treatment

The sutures were removed 2 weeks postoperatively, after the incision had healed. After the operation, the shoulder joint was passively exercised, while active motion of the shoulder was initiated approximately 4–6 weeks later. After 9–10 weeks of postoperative X-ray bone healing, the shoulder joint was subjected to anti-resistance exercise and active activities. There were no differences in postoperative activity regimens between the two groups.

### Data collection

We collected data on the patient’s sex, age, operation time, blood loss, and wound healing time. One year after operation, the Constant–Murley score [20] was used to evaluate the shoulder joint function. The total score for this system is 100 points, with 15 points for pain, 20 points for activities of daily living, 40 points for range of motion, and 25 points for strength. A score of 90–100 points shows excellent shoulder joint function; 80–89 points, good; 70–79 points, moderate; and < 70 points, poor. Pain symptoms were assessed using the pain visual analog scale. The “excellent” and “good” rate were calculated as follows: (number of people with excellent + good score)/total number of people × 100%. Radiographic images and computed tomography scans were taken regularly at 1, 3, 6, and 12 months after surgery to monitor the fracture healing and to determine whether there was avascular necrosis of the humeral head, infection, or screw penetrations. The results were evaluated by five surgeons.

### Statistical analyses

Statistical analysis was performed using SPSS 18.0 software. All data are presented as the mean values ± standard deviations. Differences between the groups were tested using Student’s t-test. Binary variables were assessed using the χ^2^ test. Post hoc power analysis was performed with the G-power tool using an alpha error of 0.05. *P*-values < 0.05 indicated statistical significance.

## Results

From June 2012 to June 2017, 317 patients with OTA/AO type 11C1.1 and 11C3.1 proximal humerus anatomic neck fractures were admitted to our hospital. Of these, 68 patients were included in the present study after application of the inclusion and exclusion criteria. All patients with OTA/AO type 11C1.1 and 11C3.1 proximal humerus anatomical neck fractures were treated with internal fixation surgery, and there were no patients with hemi-shoulder replacement or total shoulder replacement. Both subtypes 11C1.1 and 11C3.1 were equally treated with locking plate or intramedullary nail fixation.

Overall, we enrolled 68 patients aged 38–85 years (mean 66.5 years), of whom 21 were males and 47 were females. This cohort included 43 cases of OTA/AO type 11C1.1 and 25 cases of OTA/AO type 11C3.1. Both subtypes 11C1.1 and 11C3.1 were equally treated with locking plate or intramedullary nail fixation. A total of 35 patients underwent open reduction and locking plate internal fixation, and 33 patients underwent limited open reduction and intramedullary nail internal fixation. In the locking plate group, six patients with severe osteoporosis or medial wall crushing, which could not be reconstructed, underwent bone grafting, including two cases of allograft fibula transplantation and four cases of allograft iliac bone transplantation. After reduction, these six patients chose Wright artificial bone to fill the defect. As the intramedullary nail is centrally fixed and maintains good alignment of the fracture without the need for bone grafting, no patients required bone grafting. The patient characteristics are shown in Table 1. The two groups were comparable, with no significant differences in age, sex, or fracture type (*P* > 0.05).

**Table 1:** Comparison of the patients’ basic characteristics

Group	Locking plate group (n = 35)	Intramedullary nail group (n = 33)	*χ* ^*2*^ */t*	*P*-value
Age (years)	66.5 ± 5.8	64.1 ± 6.9	1.556	0.125
Sex (n)			0.010	0.920
Male	11	10		
Female	24	23		
Fracture type			2.083	0.209
11C1.1	25	18		
11C3.1	10	15		

The mean follow-up time for all patients was 17.8 months (range, 9–26 months). The mean operation time of the locking plate group (95.3 ± 12.5 min) was significantly longer (*P* < 0.05) than that of the intramedullary nail group (75.9 ± 10.3 min). Moreover, the mean bleeding volume of the locking plate group (200.6 ± 23.3 ml) was significantly higher (*P* < 0.05) than that of the intramedullary nail group (100.3 ± 20.4 ml). The wound healing time of the intramedullary nail group was shorter than that of the locking plate group, but the difference between the two groups was not statistically significant (*P* > 0.05, Table 2).

**Table 2:** Follow-up results of the intramedullary nail group and the locking plate group

Parameter	Locking plate group (n = 35)	Intramedullary nail group (n = 33)	*t/χ*^*2*^ value	*P-*value
Healing time (months)	3.3 ± 1.2	3.1 ± 1.9	0.687	0.494
Initial neck shaft angles (°)	137.5 ± 7.8	136.4 ± 6.9	0.615	0.541
Last neck shaft angles (°)	133.3 ± 6.13	134.5 ± 7.21	0.741	0.461
Forward lift range (°)	143.9 ± 20.36	139.6 ± 21.23	0.853	0.397
Shoulder joint score (°)	79.8 ± 8.1	81.9 ± 7.6	-1.101	0.275
“Excellent” and “good” rate (%)	82.9	86.7	0.342	0.716

The initial and final neck–shaft angles of patients in the locking plate group were 136.4° ± 6.9° and 134.5° ± 7.21°, respectively, and the average degree of decline was 1.9°. The initial and final neck–shaft angles of patients in the intramedullary nail group were 136.4° ± 6.9° and 134.5° ± 7.21°, respectively, and the average degree of decline was 1.9°. The initial and final neck–shaft angles showed no significant differences between the two groups (*P* > 0.05). The mean forward flexion range of the locking plate group was 143.9° ± 20.36°, while that of the intramedullary nail group was 139.6° ± 21.23°, with no significant differences between the two groups (t = 0.853, *P* > 0.05). In addition, we found no statistical difference in the mean values of the Constant–Murley scores or “excellent” and “good” rates between the locking plate and intramedullary nail groups (*P* > 0.05, Table 2).

Complications (including screw penetrations, acromion impingement syndrome, infection, and aseptic necrosis of the humeral head) occurred in 8 patients (8/35, 22.8%) in the locking plate group, all of whom had varus fractures of the proximal humerus. Two patients in the locking plate group showed screw penetrations. Further, three patients developed acromion impingement syndrome, including one patient with high plate placement accompanied by postoperative displacement of the greater tuberosity humerus, and one patient with postoperative redisplacement of the greater tuberosity humerus. One patient developed an infection three months after surgery. The infection was controlled after the steel plate was removed, flushed, and drained. Two patients developed aseptic necrosis of the humeral head. Conversely, complications (including malunion and acromion impingement syndrome) occurred in 5 patients (5/33, 15.1%) in the intramedullary nail group, all of whom had valgus fractures of the proximal humerus. Two patients in the intramedullary nail group had malunion, and three patients had acromion impingement syndrome. There was no significant difference in the number of complications between the two groups (*P* > 0.05).

## Discussion

The treatment modalities for anatomic neck fractures include internal fixation, hemi-shoulder replacement, and reverse shoulder replacement, while the surgical options of internal fixation predominantly include locking plates and intramedullary nailing [21–24]. The treatment modality of choice is however influenced by factors including age, fracture type, and functional status. Recently, the minimally invasive plate osteosynthesis (MIPO) technique for proximal humerus fractures has been indicated to provide good clinical results as an alternative to standard open plating [25, 26]. Previous studies have shown that the MIPO technique provides satisfactory clinical results with few complications for proximal humerus fractures [27, 28]. However, MIPO is technically demanding for the proximal humerus because the technique reduces the fracture in a closed fashion, which prevents both direct visualization of the fracture and implant manipulation. In addition, the MIPO technique may result in malalignment and malrotation problems due to improper fracture reduction [29]. Therefore, for proximal humerus fractures (especially comminuted proximal humerus fractures), surgeons prefer the incisional approach to internal fixation. The incisional approach allows direct visualization of the fracture reduction and thus ensures its reliability. In our study, to compare the clinical efficacy of intramedullary fixation and locking plate fixation for OTA/AO type 11C1.1 and 11C3.1 proximal humerus anatomical neck fractures, we retrospectively analyzed the follow-up data of two surgical groups. Our analysis showed that the operation times of the locking plate group were significantly longer than those of the intramedullary nail group. Similarly, the postoperative bleeding volumes of the locking plate group were significantly higher than those of the intramedullary nail group. The wound healing times, Constant–Murley scores, and “excellent” and “good” rates, in contrast, showed no significant differences between the two groups. Moreover, there was no statistical significance in the number of complications between the locking plate and intramedullary nail groups.

For proximal humerus fractures, locking plates remain the gold standard internal fixation method, the indications for which include fractures of the proximal humerus; however, the complication rate of using locking plates remains high. Wu et al. [30] summarized the data of 514 patients with proximal humerus fractures treated with locking plates, and found that the overall complication rate was 48.8%, including a varus displacement rate of 16.3% and avascular necrosis of the humeral head rate of 10.8%. In the locking plate group, there were two cases of screw penetrations, three cases of acromion impingement syndrome, and two cases of aseptic necrosis of the humeral head, all of which were varus fractures of the proximal humerus. For OTA/AO type 11C3.1 proximal humerus fracture of patients, where the humeral head and stem are not fully repositioned, there is always a significant risk of varus displacement, which will inevitably lead to the difficulty of reduction and fixation of the greater tuberosity. Moreover, the varus traction stress generated by the rotator cuff and the combined medial humerus calcar comminution led to varus collapse and treatment failure, which eventually led to humeral head necrosis. All patients with complications in the locking plates developed varus fractures of the proximal humerus. Gregory et al. [31] concluded that the probability of humeral head necrosis is related to the quality of repositioning and the biomechanical properties of fixation, as opposed to the size of the initial fracture displacement. Measures to avoid varus fractures of the proximal humerus include neutralizing rotator cuff tension, repairing and reconstructing the medial humeral spacing, and enhancing bone grafting. Moreover, allograft fibula or femoral head implants can effectively prevent re-inversion of the humeral head in patients with severe osteoporosis and those at an advanced age [32, 33]. In the present study, the use of locking plates to fix valgus fractures of the humerus was associated with few complications. The fracture site of patients in the locking plate group predominantly occurred on the lateral side, ensuring that satisfactory repositioning and internal fixation could be obtained through the lateral plate, as long as the humeral head was elevated to restore the neck–shaft angle of the humerus and was supported by bone grafting. Higher placement of the plate and postoperative redisplacement of the greater tuberosity are related to the surgical technique used. After completion of plate fixation, the rotator cuff fixation must be strengthened with sutures, especially in patients with comminuted fractures of the greater tuberosity. Moreover, it is necessary to strengthen the fixation to prevent displacement of the greater tuberosity.

The complication rate of the intramedullary nail group was 15.1% (5/33), and complications occurred only in patients with valgus proximal humerus fractures. Among them, there were two cases of malunion and three cases of acromion impingement syndrome (including one case of screw tail exposed and two cases of postoperative redisplacement of the greater tuberosity humerus). These patients had poor postoperative function, which may be related to the reduction and fixation of the greater tubercle. In adduction fractures, the medial cortex is more severely damaged, and the stress concentration of the intramedullary nail is less than that of the laterally fixed steel plate. Compared with angled locking plates, intramedullary nails have higher mechanical strength. This higher strength can reduce the dissection of soft tissue at the fracture site, preserve the blood supply to the fracture, and facilitate fracture healing. For patients with comminuted greater tuberosity fractures accompanied by anatomical neck fractures, if intramedullary nails are to be used for fixation, the Wright artificial bone can be used following reduction of the humeral head to restore the thickness of the greater tuberosity and to promote the restoration of rotator cuff tension.

This study has a few limitations. First, this study had a retrospective cohort study design, with a small number of patients and a limited level of evidence. As such, further studies are needed to validate the results. Second, although all procedures were performed by two surgeons who were equally proficient in both methods, it is possible that there were some differences in the way these two surgeons performed the procedure, potentially leading to some bias.

## Conclusion

Our assessment did not show any significant differences in wound healing times, forward flexion ranges, Constant–Murley scores, “excellent” and “good” rates, and complications between the locking plate and intramedullary nail groups. However, our results indicated that using intramedullary nails has an advantage over locking plates in terms of reduced operation time and bleeding volume. However, this is only a preliminary study, and larger trials are required to verify the results of this study in the future.

## Data Availability

The data used for analysis in this study are available from the corresponding author upon reasonable request.
